# Implant Stability Changes for a Single Implant Mandibular Overdenture

**DOI:** 10.1055/s-0041-1736416

**Published:** 2021-12-08

**Authors:** Karim Fouda, Ahmed Fahmy, Khaled Aziz, Marwa Abdel Aal, Amr Naguib, Nouran Abdel Nabi

**Affiliations:** 1 Department of Prosthodontics, Faculty of Dentistry, Cairo University, Cairo, Egypt

**Keywords:** nonsubmerged, Periotest, single-implant retained mandibular overdenture, submerged

## Abstract

**Objectives** To compare the changes in implant stability for the nonsubmerged and submerged protocols for a single-implant retained mandibular overdenture using Cendres and Metaux Locator attachment throughout a 24-month follow-up.

**Materials and Methods** Eighty edentulous patients who were seeking to install a single implant in the midline of the completely edentulous mandible. At the day of implant installation, patients were randomized into two groups using sealed envelopes: the nonsubmerged and submerged groups. After 3 months of healing period, randomization using sealed envelopes was performed and patients were randomized to receive the Cendres and Metaux Locator attachment. The periotest readings were recorded using the Periotest M device, every 3 months for the first year and annually in the second year. The scope of this clinical trial focused only on results of the Cendres and Metaux attachment.

**Statistical Analysis** The Mann–Whitney *U* -test was used for comparison between study groups for independent samples. Two-sided *p* -values less than 0.05 were considered statistically significant.

**Results** There was no statistically significant difference between the mean periotest readings of both groups throughout the 24-month follow-up. Both groups showed an improvement in mean periotest readings with the submerged group tending to show greater stability at 6, 12, and 24-month follow-ups.

**Conclusions** The nonsubmerged and the submerged healing protocols resulted in reliable periotest readings with the submerged group showing greater improvement than the nonsubmerged, although this improvement is nonsignificant when using the Cendres and Metaux attachment for a single mandibular overdenture.

## Introduction

Brånemark et al in 1969 first reported the successful outcomes of the submerged surgical procedure in implant dentistry. 1 The submerged surgical protocol enhanced the process of new bone formation and remodeling by utilizing a two-stage surgical procedure. 2 The two-stage surgical protocol proved good short- and long-term outcomes. 3

On the other hand, osseointegration was successfully achieved through a single-stage “nonsubmerged surgical protocol,” in which implants and the healing abutment were exposed in the oral cavity during the period of osseointegration. 4 The nonsubmerged surgical protocol offered several advantages: it requires only a single-stage surgery which was more cost-effective, 5 reducing postoperative complications, with no micro-gap at the alveolar bone crest level. 6 On the other hand, the submerged surgical technique was indicated in almost all cases, specifically in cases where bone augmentation was required to ensure optimum healing. 7

Primary implant stability was mainly associated with the mechanical engagement of the implant to the surrounding bone, whereas bone generation and remodeling phenomena determine the secondary (biological) stability. 8 Bone quantity, bone quality, surgical technique, and implant design are factors that influence primary stability, while primary stability, bone remodeling, and implant surface conditions are considered as important factors that will influence secondary implant stability.

The Periotest device and resonance frequency analysis using the Osstell device were considered as noninvasive methods to measure implant stability. 9 Primary and secondary implant stability measurements using both devices resulted in reproducible quantitative values. The periotest is an electronic instrument designed to give quantitative measurements of the damping characteristics of the periodontal ligament surrounding a tooth, thus establishing a value for its mobility. 10 The software in the instrument is designed to relate contact time as a function of tooth mobility. The result is displayed as periotest values (PTVs) on a scale of −8 (low mobility) to 50 (high mobility). A stable implant will exhibit different stiffness characteristics compared with those of teeth that are connected by a periodontal ligament.

The McGill consensus (2002) and York consensus (2009) stated that two implants installed in the mandible was considered to be the standard of care for completely edentulous patients. 11 12 Harder et al and Cheng et al proved that a single implant installed in the midline can be an efficient treatment option as two implants installed in the mandible. 13 14 Cordioli et al introduced the idea of installing a single implant in the midline of a completely edentulous mandible to retain an overdenture. 15 The single-implant retained mandibular overdenture is considered to be a cost-effective treatment option which proved medium- to long-term survival rates. 16 17 18 19

The choice of the attachment system for the implant-retained overdentures was considered to be of great importance as it has an impact on the overall patient satisfaction and the clinical success. 20 A newly introduced attachment made from polyetherketoneketone (PEKK) which is a member of the polyaryletherketones (PAEKs), is currently in use. PAEKs have the advantage of high chemical and mechanical resistance to wear and high tensile, fatigue, and flexural strengths. 21 According to the manufacturer Cendres and Metaux, PEKK has 80% higher compressive strengths than other PAEK materials. Passia et al, Choi et al, and Maniewicz et al concluded that the combination of a titanium patrix and a matrix made from PEKK seems to be a promising combination for long-term retention, on parallel and axillary tilted implants. 22 23 24

The aim of this randomized clinical trial was to compare the changes in implant stability using periotest device for the nonsubmerged and submerged protocols for the single-implant retained mandibular overdenture using Cendres and Metaux Locator (CM-LOC) attachment for a 24-month follow-up.

## Materials and Methods

The study proposal was approved by the ethical committee on June 13, 2016 (ethical approval No. 16/6/10) and is registered at http://www.pactr.org/ (trial PACTR201803003085193). The guidelines of the World Medical Association were implemented in this clinical trial.

Eighty completely edentulous patients were recruited following a strict inclusion criteria. All patients received a single implant in the midline of the edentulous mandible. At the day of implant installation, patients were randomized using sealed envelopes into two groups: nonsubmerged (NS) and submerged (S); a 3-month healing period was allowed for all patients in both groups. The present study has followed the same inclusion criteria, sample size calculation, and all of the clinical relevant procedures as the trail performed by AAl et al. 25 All included patients (age range: 50–69 years) were recruited following strict inclusion criteria: patients with glycosylated hemoglobin level ≥8, patients seeking to install a single median implant in the mandible, and for whom new dentures will be constructed were included. Patients with any condition that would contraindicate implant placement were excluded.

All patients received ZDI implants with a tapered screw vent (Zimmer Dental, Warsaw, Indiana, United States), with a diameter of 3.7 mm and a length of 10 mm. Drilling was performed using the Zimmer Dental kit following the manufacturer's instructions.

This clinical trial followed up the changes in periotest readings (PTV) for the NS and S groups after the 3 months of healing period (day of pick-up), after which second randomization was followed.

### Patient Distribution after 3 Months of Healing Period (Day of Pick-Up)

**Fig. 1:**
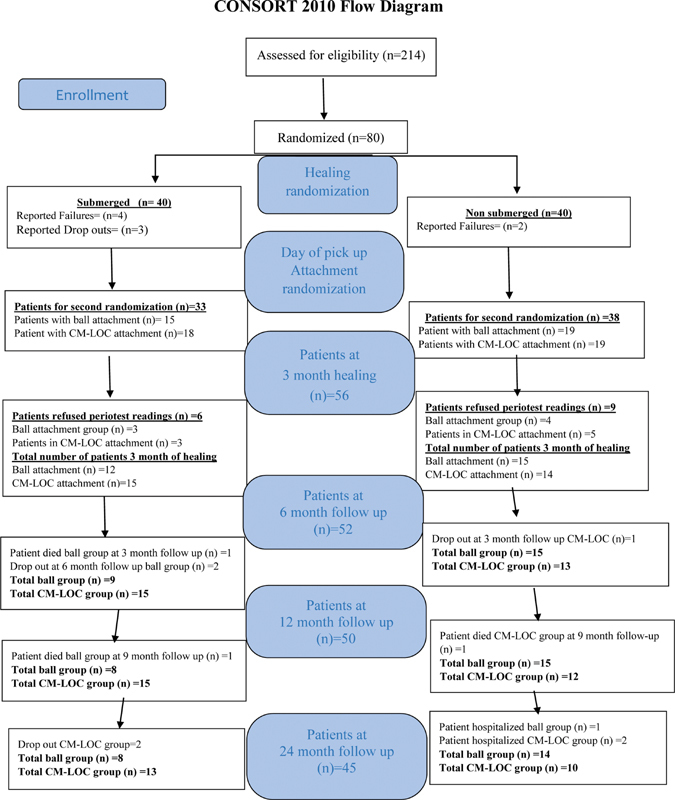
Consort flow diagram showing the number of included patients and drop-outs during 24-month follow-up period.

During the 3-month healing period, four patients reported failure and three patients were counted as drop-outs from the S group. While for the NS group, two patients reported failure ( Fig. 1 ). The number of patients who were recalled after the 3-month healing period was 71; 33 patients in the S group and 38 patients in the NS group.

Patients were randomized after healing using sealed envelopes to receive CM-LOC attachment. The distribution of patients in each of the groups is described in Table 1 . Randomization and allocation concealment were performed by A.N., as he was responsible for preparing the envelopes used in randomization.

**Table 1:** Distribution of patients throughout the follow-up intervals for all groups of patients

	3 months after healing	Submerged		Nonsubmerged	
		Ball group	CM-LOC group	Ball group	CM-LOC group
Number of patients	71	15	18	19	19
Number of males	50	11	14	11	14
Number of females	21	4	4	8	5
Mean age of males	60.4 y	61.8 y	59.3 y	59.1 y	61.7 y
Mean age of females	60.8 y	64.75 y	58.5 y	63.1 y	57.2 y
Number of patients refused periotest reading		3 patients 2 males 1 female	3 patients All males	4 patients All males	5 patients 2 males 3 females
Number of drop-outs at 3-month follow-up		1 patient died 1 male	–	–	1 patient 1 male
Number of drop-outs at 6-month follow-up		2 patients 1 male 1 female	–	–	–
Number of drop-outs at 9-month follow-up		1 patient died 1 male	–	–	1 patient died 1 male
Number of drop-outs at 12-month follow-up		–	–	–	–
Number of drop-outs at 24-month follow-up		–	2 patients All males	1 patient (hospitalized) 1 female	2 patients (hospitalized) All males

### Intervention

At the day of pick-up the healing abutments in the NS group were unscrewed and CM-LOC attachment was screwed in place with a torque of 30 N-cm. While for the S group a small crestal incision was made at the area corresponding to the attachment, and then the attachment was screwed.

**Fig. 2:**
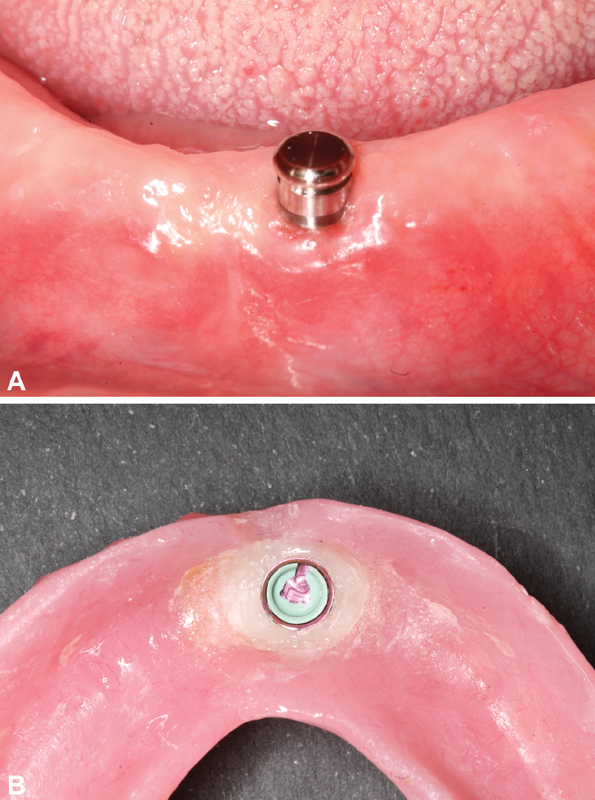
( **A** ) CM-LOC attachment screwed inside the patient's mouth. ( **B** ) The PEKK green cap of the CM-LOC attachment after picked up in the fitting surface of the denture. CM-LOC, Cendres and Metaux Locator.

The CM-LOC attachment system comprises a male implant straight abutment with a gingival cuff height ranging from 1 to 5 mm ( Fig. 2A ). The housings are made either of PEKK (Pekkton; Cendres + Métaux SA) or of titanium, and lodge a Pekkton retentive insert available in different strengths; in this study the “medium” (green) retentive insert was used.

After the attachments were screwed in place, the patient was instructed to sit in an upright position. The Periotest M (Medizintechnik Gulden e.K., Modautal, Germany) was directed to the midbuccal surface perpendicular to the long axis of the screwed attachment where the tapping rod was directed at the bottom of an attachment. To measure the implant stability (periotest reading, damping effect = PTV) at the day of pick-up (3 months after healing), which was considered to be the baseline reading, five readings were recorded for each patient, and then the average reading was recorded in the patient's file ( Fig. 2B ). A.F. and K.F. were responsible for recording the PTV readings for all groups of patients throughout the 24-month follow-up period.

The CM-LOC attachment was screwed to the implant with a torque of 30 N-cm, with the corresponding matrix on top of it (PEKK matrix). The mandibular denture was modified and then checked for proper seating, and the occlusion with the maxillary denture was properly checked. A soft mix of Luxa pick-up material (DMG) then added to the modified denture, and the patient was then asked to close in centric occlusion. After complete setting of the Luxa pick-up, the denture was removed and the pick-up of the matrix was checked ( Fig. 2B ).

The periotest reading (PTV) was recorded for all patients (as in baseline, five readings with on average), recorded every 3 months for the first 12 months, and then annually at 24-month follow-up.

After 3-month healing, 33 patients were present in the S group, and 38 patients in the NS group, while at 24-month follow-up, 45 patients attended the 24-month follow-up ( Fig. 1 and Table 1 ).

Data were statistically described in terms of mean ± standard deviation. Comparison of numerical variables between the study groups was done using Mann–Whitney *U* -test for independent samples. Two-sided *p* -values less than 0.05 were considered statistically significant. All statistical calculations were done using the computer program IBM SPSS (Statistical Package for the Social Science; IBM Corp, Armonk, New York, United States) release 22 for Microsoft Windows.

## Results

The inter-observer consistency for the two readings of the PTV was recorded by A.F. and K.F. for both groups (NS and S) during different follow-up intervals using Cronbach's α statistics ( Table 2 ). Results of the inter-observer consistency showed a strong agreement for both groups at the different follow-up periods, as the values at the different follow-up periods for both groups were greater than 0.7.

**Table 2:** Inter-observer consistency using Cronbach's α statistics

	3 months after healing	3-month follow-up	6-month follow-up	9-month follow-up	12-month follow-up	24-month follow-up
Cronbach's α NS group	0.965	0.991	0.995	0.969	0.975	0.953
Cronbach's α S group	0.946	0.981	0.997	0.957	0.995	0.979

Abbreviations: NS, nonsubmerged; S, submerged.

### Comparison of Mean Periotest Readings (PTV) between the NS and S Groups for CM-LOC Attachment at Different Follow-Up Intervals

**Fig. 3:**
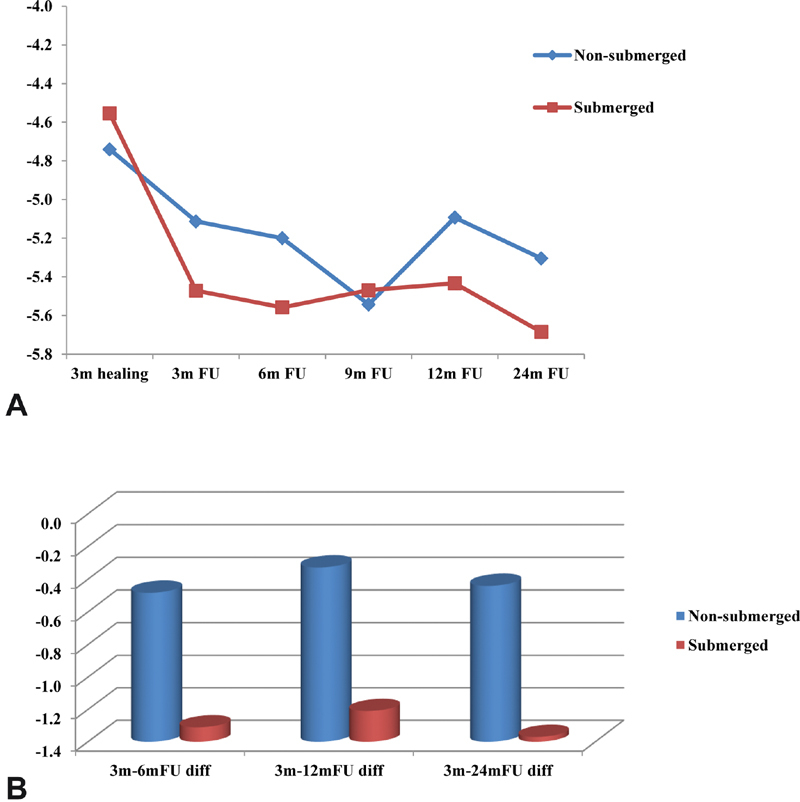
( **A** ) Mean PTV between the nonsubmerged (NS) and submerged (S) groups of patients with CM-LOC attachment. ( **B** ) Mean PTV changes between the nonsubmerged (NS) and submerged (S) groups of patients with CM-LOC attachment. CM-LOC, Cendres and Metaux Locator.

There was no statistically significant difference between the mean PTV readings of the NS and S groups for CM-LOC attachment throughout the 24-month follow-up. At baseline, the NS group recorded higher mean PTV readings than the S group (−4.740 ± 0.7414, −4.555 ± 0.8676, *p* = 0.639; Table 3 and Fig. 3A ). Starting from 3-month follow-up, the mean PTV readings for the S group were greater than those for the NS group (−5.11 ± 0.866, −5.47 ± 1.219, *p* = 0.190) and continued till 24-month follow-up, with being nearly equal at the 9-month follow-up period ( Table 3 and Fig. 3A ).

**Table 3:** Mean and standard deviation for the nonsubmerged (NS) and submerged (S) groups of patients with CM-LOC attachment

Group	3 mo (baseline)	3-month follow-up	6-month follow-up	9-month follow-up	12-month follow-up	24-month follow-up
NS	−4.740 ± 0.7414	−5.11 ± 0.866	−5.2 ± 1.1173	−5.543 ± 0.9177	−5.093 ± 0.9177	−5.304 ± 0.8092
S	−4.555 ± 0.8676	−5.47 ± 1.219	−5.558 ± 0.8649	−5.469 ± 0.7867	−5.433 ± 1.1926	−5.685 ± 0.8245
*p* -Value a	0.639	0.190	0.429	0.661	0.205	0.276

Note: Numbers are presented as mean and standard deviation.

a *p* -Value ≤0.05 is considered statistically significant.

### Changes in Mean Periotest Readings (PTV) between the NS and S groups for CM-LOC Attachment at Different Follow-Up Intervals

There was an improvement in the changes of mean PTV readings for the CM-LOC attachment in the first 6, 12, and 24 months from baseline; it was clear that this improvement was revealed for both the NS and S groups with no statistically significant differences between them ( Table 4 ). The S group showed greater improvement in the mean PTV values when compared with the NS group at the first 6, 12, and 24 months, showing the greatest improvement at 24-month follow-up (−0.441 ± 1.1320, −1.370 ± 1.2093, *p* = 0.192) ( Table 4 and Fig. 3B ).

**Table 4:** Mean and standard deviation for the nonsubmerged (NS) and submerged (S) groups of patient with CM-LOC attachment

Group	3-month healing–6-month follow-up	3-month healing–12-month follow-up	3-month healing–24-month follow-up
NS	−0.483 ± 1.2305	−0.329 ± 1.1913	−0.441 ± 1.1320
S	−1.311 ± 1.2995	−1.210 ± 1.4106	−1.370 ± 1.2093
*p-* Value a	0.213	0.095	0.192

a *p* -Value ≤0.05 is considered statistically significant.

## Discussion

Implant stability is one of the important parameters that would influence the successful osseointegration of dental implants. Primary implant stability is the mechanical stability, whereas secondary implant stability is the biological phenomenon and is the result of osseointegration. Several methods were used to measure primary and secondary implant stability, but RFAs using Osstell and Periotest are the most noninvasive methods commonly used to objectively monitor implant stability at different observation periods. 26 In the present study, the periotest was used to monitor the changes in secondary implant stability, because the Osstell would require the smart peg to be attached to the implant and that would require unscrewing of the attachment each time during measurement, so it was not applicable. Zix et al proved that the periotest was more user-friendly and time- and cost-efficient because the superstructure should not be removed while performing the measurments. 27 Despite the fact that both instruments are used to evaluate stability, Meredith et al reported that the periotest had low reproducibility and sensitivity, 28 while on the other hand several studies concluded that the periotest was a reliable method to objectively determine implant stability 12 29 ; furthermore Khalaila et al concluded that the periotest was a reliable tool for assessing implant stability and it would provide predictive information about marginal bone loss. 30

Inter-operator and inter-instrument variability was considered to affect the periotest scores, such as the angulation and positioning of the device hand piece (horizontal distance and angle of the implant). In the following trial, the periotest was held as a “pen grip” in the anterior area, being perpendicular at the midbuccal area of the attachment as was described in the periotest user manual by Schulte and Lukas. 31 It was concluded that a single periotest measurement will not allow prognosis for the stability of an implant, and so that was the reason that five readings were recorded for each patient, and an average reading was then recorded in the patient's file.

The bone quality and quantity are important factors that influence the primary implant stability. The more dense the bone the better the initial stability. 32 In the present study, all implants were ZDI implants with the same diameter and length and were installed in the midline of the anterior mandible which is considered to be of dense bone as classified by Lekholm and Zarb, 33 so all of the installed implants in both the S and NS groups were of high initial stability with a mean PTV reading ranging from −4.4 to −5.8. Olivé and Aparicio further confirmed that the PTV readings of dental implants lie between a narrow zone of −5 to +5, where −5 was considered to be of high stability. 29 The initial stability will consequently influence secondary implant's stability, 34 this was the reason why the PTV readings recorded in both the S and NS groups after 3 months of healing were of high secondary stability, as the more negative values of the periotest indicate greater implant stability. 35 Truhlar et al concluded that the PTV at second-stage surgery is the best estimate for the bone–implant contact (BIC), as PTV determines implant stability and more specifically BIC which was mainly influenced by bone quality. 36

The CM-LOC attachment was used in several *in vitro* studies, and proved to record to show consistent values of retention even with tilted implants. 22 23 24 As clinical studies are always important to confirm conclusions, it was difficult to measure retention inside the patient's mouth. So in the present study, the changes in stability for the NS and S groups using CM-LOC attachment were reported.

There was an improvement in PTV readings for the NS and the S group from the baseline (day of pick-up) till 24 months of follow-up without any significant difference between the groups. The PTV readings for the NS group were initially greater at baseline (day of pick-up) when compared with the S group, then starting from 3-month follow-up till 24-month follow-up, the S group showed greater PTV when compared with NS. An explanation for this is that during the 3-month healing, the NS group had a healing abutment and the fitting surface of the denture was relieved by applying a soft liner to help reduce the forces on the installed implant for successful osseointegration. The NS group was subjected to more mechanical stimulation than the S group, this mechanical stimulation enhanced bone formation in the NS than the S group. 37 Branemark et al reported that new bone formed under loading conditions 38 consisted mainly of mature lamellar bone which is of greater density compared with the new bone formed under unloaded conditions, this phenomena was referred to as “form follows function,” so initially the NS group had higher PTV readings than the S group. 39 However, for the S group, the PTV readings started to show greater scores after the pick-up and loading of the attachments which resulted in physiologic mechanical stimulation that consequently led to mature lamellar bone formation and thus greater BIC which consequently improved the PTV readings over 24-month follow-up. It is clear from the present study that at 6-, 12-, and 24-month follow-ups, the S group showed a greater improvement in the mean change in PTV readings than the NS group, which is in agreement with Levy et al who reported through histomorphometric analysis that BIC is greater for the submerged protocol. 40

## Conclusion

Both the nonsubmerged and the submerged healing protocols resulted in reliable periotest readings using CM-LOC attachment for a single-implant retained overdenture. The S group showed a greater improvement change in periotest readings after 12- and 24-month follow-up periods when compared with the NS group, although this improvement was not statistically significant.
